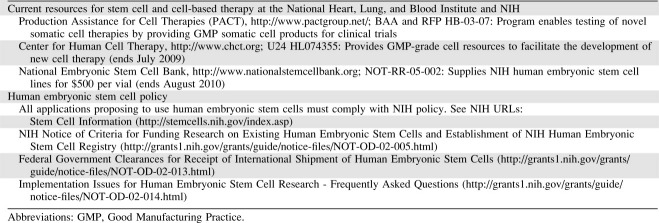# National Heart, Lung, and Blood Institute Perspective: Lung Progenitor and Stem Cells—Gaps in Knowledge and Future Opportunities

**DOI:** 10.1002/stem.148

**Published:** 2009-06-11

**Authors:** Carol J Blaisdell, Dorothy B Gail, Elizabeth G Nabel

**Affiliations:** National Heart Lung and Blood Institute, National Institutes of HealthBethesda, Maryland, USA

**Keywords:** Lung cell biology, Lung regeneration, Reprogramming

## Abstract

Because the lung stem cell field is so new, there remain many unanswered questions that are being addressed regarding the identification, location, and role of exogenous and endogenous stem and progenitor cell populations in growth, regeneration, and repair of the lung. Advancing lung stem cell biology will require multidisciplinary teams and a long term effort to unravel the biologic processes of stem cells in the lung. While no clinical research in lung stem cell therapies are currently funded by NHLBI, the knowledge gained by understanding the basic biology of the lung stem cell populations will be needed to translate to diagnostic and therapeutic strategies in the future.

## INTRODUCTION

In the new millennium, research on lung stem/progenitor cell biology and cell-based therapies for regeneration and repair of lung disease is of great interest not only to scientists but also to the public. The field of lung stem/progenitor cell biology is in its infancy, particularly compared with studies of other continuously renewing cell populations such as skin and gut stem cells, and there are many opportunities for important progress and future discovery. Our goal in writing this brief perspective is to highlight some of the current issues and future opportunities and challenges in the field and to provide information on resources and new initiatives in progenitor and stem cell biology supported by the National Heart, Lung, and Blood Institute (NHLBI).

**Figure 1:**
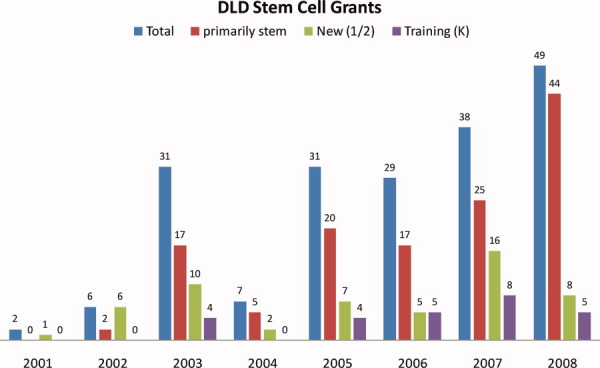
Number of National Heart, Lung, and Blood Institute-supported grants in lung stem/progenitor cell biology (2001-2008). New refers to new applications (type 1 or 2) to the NIH. Abbreviation: DLD, Division of Lung Diseases.

Since 2001, when the grant portfolio of the NHLBI in lung progenitor/stem cell biology consisted of less than a handful of projects, the program has grown to almost 50 such grants in fiscal year 2008 (Fig. 1). These research studies are examining several aspects of lung stem cells such as the pluripotency of lung resident cells during development and after lung injury, the impact of mesenchymal stromal cells in preclinical studies of acute lung injury, and the potential to correct specific genetic defects that impact lung host defenses in lung diseases such as cystic fibrosis. Because the lung stem cell field is so new, there remain many unanswered questions that are being addressed regarding the identification, location, and role of exogenous and endogenous stem and progenitor cell populations in growth, regeneration, and repair of the lung [1–5]. More than a decade ago Emura suggested “what is urgently required is, therefore, to establish unequivocal markers for the identification and isolation of respiratory stem cells” [6], but these markers are still undefined.

**Figure 2:**
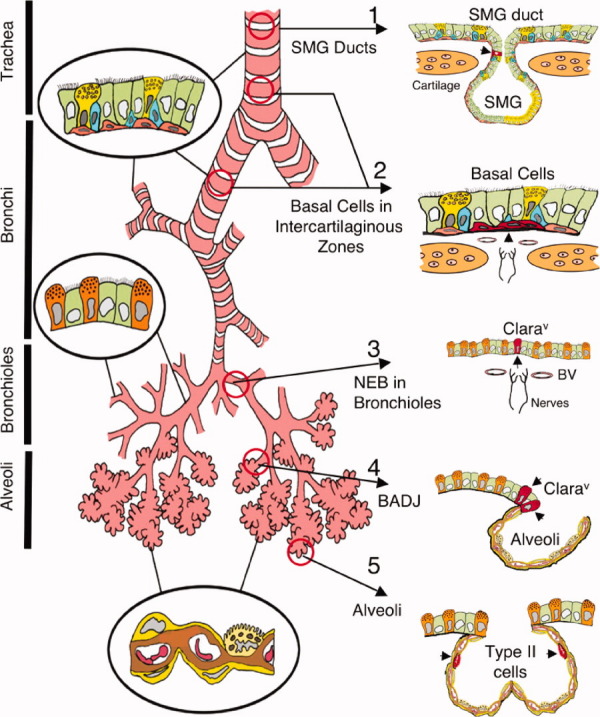
Illustration of putative stem cell niches in the adult mouse lung. Epithelia of the adult mouse lung can be divided into four major, biologically distinct trophic units (trachea, bronchi, bronchioles, and alveoli), each of which encompasses unique types of airway epithelial cells (epithelia relevant to each unit are shown inside circles). Five potential stem cell niches for these various trophic units are shown on the right, with locations of candidate stem cells marked by arrowheads (cells are in red). Stem cells and niches include the following: 1, an unknown cell type in the SMG ducts of the proximal trachea; 2, basal cells in the intercartilaginous zones of the lower trachea and bronchi (these structures may also be associated with innervated NEBs; 3, variant Clara cells associated with NEBs in bronchioles; 4, Clara cell associated with BADJ; and 5, alveolar type II cells of the alveoli. Abbreviations: BADJ, bronchiolar alveolar duct junctions; Clara^v^, variant Clara cells; NEB, neuroendocrine body; SMG, submucosal gland. (From Liu X, Engelhardt JF. The glandular stem/progenitor cell niche in airway development and repair. Proc Am Thorac Soc 2008;5:682-688.)

Unlike the hematopoietic stem cell hierarchy, little is known about specific cells in the lung that have the capacity to self-renew, the organization of stem/progenitor cells in the lung and whether it conforms to a classic or a nonclassic stem cell hierarchy, and the differentiation pathways for the more than 40 phenotypically and functionally distinct types of cells that are required for air to be conducted to alveoli and transfer of oxygen and carbon dioxide into and out of the blood. There are at least five putative epithelial stem/progenitor cell niches in the adult mouse airway [7] (Fig. 2), as well as endothelial stem cells in the pulmonary vasculature and airway smooth muscle stem cells. In addition, many cells have host defense properties that are needed to protect the lung against inhaled particles of pollutants, microorganisms, and antigens. The human lung has 26 branches of airways that lead to alveoli with a surface area for gas exchange of 100 m^2^. Repair after injury to different regions of the lung is expected to require different populations of stem cells for regeneration. As airways develop, there is close proximity of the developing pulmonary vascular system and cross-talk between endothelial precursors and the epithelium for normal lung development to occur [8,9]. As with the pulmonary epithelium, different cellular subpopulations of the pulmonary endothelium probably have distinct embryonic origins.

## LESSONS LEARNED FROM LUNG DEVELOPMENT

Lung structure programming is incredibly reproducible in the developing mammalian embryo and fetus, which should permit identification of resident embryonic stem/progenitor cell populations with techniques such as lineage tracing and live imaging. Metzger et al. [10] recently demonstrated that the airway branching process is remarkably stereotyped during prenatal development. The airway tree is established using combinations of three geometrically simple modes of branching; domain branching, planar bifurcation, and orthogonal bifurcation. The processes involved in postnatal lung development of the epithelium remain to be determined. How the stem/progenitor cells in pulmonary vascular compartments participate in development, regeneration, and repair is still unknown [11]. Recent findings suggest that vascular progenitor cells may arise from endogenous vascular wall progenitors, from circulating bone marrow derived progenitors. As with the epithelial cells of the lung, the heterogeneity of pulmonary endothelial cells may require a site-specific niche of endothelial progenitors [12], or, possibly, still unknown resident endothelial progenitor cells may constitute a universal pool of progenitor cells that lack segmental specification.

Some molecular signals for the master regulator of lung development are known, but the way in which they are used to coordinate branching routines has not yet been determined. Five key signaling molecules regulate many processes in embryonic development: Wnt, fibroblast growth factor (FGF), transforming growth factor-β (TGF-β), Hedgehog, and Notch. In many different stem cell lines these signaling components are needed at some level and at some time to drive differentiation, but it is still not known what drives specific differentiation into the multiple cell types that have distinct roles in the lung.

However, significant information exists about key developmental signals for a normal lung to form. Expression of the transcription factor, thyroid transcription factor 1 (Titf-1, Ttf1, Nkx2.1), marks the lung lineage commitment in the early embryo, even before the primordial lung bud forms off the foregut endoderm. Genetic studies show that Titf1 is critical for the development of distal lung progenitors [13]. The Titf1 knockout mouse forms very abnormal lungs with insufficient differentiation for survival [14]. Studies also have shown a critical role for signaling by fibroblast growth factors in specification of the lung lineage [15]. FGF10 is expressed by lung mesenchyme and functions as a chemotactic factor during branching morphogenesis. If overexpressed, FGF10 stabilizes a progenitor cell compartment and leads to metaplastic differentiation of goblet cells [16]. In addition to maintaining epithelial progenitor cell proliferation FGF10 also coordinates alveolar smooth muscle cell formation and vascular development [17]. Retinoic acid signaling plays an important role by affecting Fgf10 expression through TGF-β, critical for expansion of lung progenitors and for formation of primary lung buds [18]. Proximal-distal patterning of the lung depends on Wnt/β-catenin signaling and is mediated, at least in part, through downstream regulation of N-*myc*, bone morphogenic protein-4, and FGF signaling [19]. Potentiation of β-catenin signaling leads to the arrested differentiation of immature bronchiolar stem cells, although β-catenin is not necessary for maintenance of the adult bronchiolar stem cell [20].

Because pulmonary vascular and distal air space growth are highly coordinated and injury to the developing lung can affect both processes [8], the role of endothelial progenitor cells in a developmental disorder, bronchopulmonary dysplasia (BPD), was examined by Balasubramaniam et al. [21]. Oxygen toxicity disrupts growth of vascular and alveolar compartments, limiting the surface area available for gas exchange, leading to BPD. Vascular endothelial growth factor (VEGF), nitric oxide, and erythropoietin in the lung contribute to mobilization and homing of endothelial progenitor cells (EPCs). After hyperoxia exposure in neonatal mice, expression of VEGF, endothelial nitric oxide synthase, and erythropoietin receptor and the number of EPCs in the blood, bone marrow, and lung were reduced. In contrast, EPCs increase in adults after hyperoxia, suggesting that there are important differences between repair responses of progenitor cells of the developing and mature lung.

## ENDOGENOUS PROGENITOR/STEM CELLS IN THE LUNG

Unlike other epithelial tissues that undergo rapid regeneration such as the gastrointestinal tract and skin, the lung has much slower turnover, which has hampered the identification of putative lung progenitor cells along with the lack of stem/progenitor cell markers and assays to identify and isolate them. Only recently, several candidate endogenous stem or progenitor cells have been identified in the trachea and lung using models of lung injury and repair. Subsets of K-14-expressing basal cells in the trachea [22] have the capacity for restoration of a differentiated epithelium after injury and are distinct from basal cells in the bronchi [23]. Scgb1a1^+^ Clara cells have the capacity to self-renew and proliferate in response to tracheal injury but are not the main source for tracheal regeneration [24].

The submucosal gland ducts in the proximal airway are suspected to contain stem cells [7]; however, little is known about glandular stem/progenitor cells and their niche. Studies suggest that the regenerating surface airway tracheal epithelium in a naphthalene injury model arises from cells migrating from gland ducts [25]. Mouse models, although useful for study of stem cell regulation and understanding the relationship between the stem cell and its niche, have some limitations for study of submucosal glands (SMGs), because the spatial distribution of cells in the proximal airway is so dissimilar from that in humans [7]. The ferret model may be more suitable because the distribution of submucosal glands in the ferret airway is much more similar to that in humans. Submucosal gland stem cells may be important for disorders such as asthma, chronic bronchitis, and cystic fibrosis, which are characterized by hypertrophy and hyperplasia of SMGs.

There is a mixed population of pluripotent cells in the lower respiratory tract that are characterized as Hoechst dye-effluxing side population (SP) cells and express molecular markers of airway and mesenchymal origin [26]. CD45^−^ SP cells isolated from human tracheobronchial epithelium have proliferative potential. Increased numbers of these cells in asthmatic airways suggest that dysregulation of pluripotent cells may play a role in the pathogenesis of this chronic disorder [27]. In the developing lung, some of these SP cells, which are CD45^+^ and CD45^−^, have endothelial progenitor cell potential to respond to hyperoxic exposure [28].

Kim et al. [29] identified a population of endogenous cells in the adult distal lung that have stem cell characteristics and coined the term bronchoalveolar stem cells (BASCs). The BASCs are resistant to naphthalene injury and proliferate in response to airway or alveolar injury. BASCs reside near bronchiolar-alveolar junctions, presumed stem cell niches. The BASCs were originally defined by expression of both CC10 and SP-C, as well as coexpression of Sca-1 and, and are capable of self-renewal and differentiation into Clara cells and alveolar cells. Hong et al. [30] identified the variant Clara cell as an endogenous lung stem cell, which expresses Clara cell secretory protein and survives naphthalene injury, unlike the more abundant progenitor Clara cell. These variant Clara cells infrequently proliferate during steady state but are felt to be responsible for repopulating the distal airway epithelium in response to injury. Reynolds et al. [31] suggested that the adult lung does not behave in a manner similar to that of blood and other organ systems that have a classic stem cell hierarchy. The lung may have a distinct biology for dealing with injury that is stem cell-independent (i.e., responds by cell proliferation, similar to the liver) using transit-amplifying progenitor cells.

The lung microcirculation is enriched in progenitor cells, but our understanding of these cells and their interrelationships is very limited. Que et al. [32] recently reported that the mesothelium overlying the lung has a progenitor population that gives rise to pulmonary vascular (but not epithelial) smooth muscle cells during embryonic development. As suggested by Stevens et al. [11], studies are needed to define the biochemical and functional phenotypes of not only the pulmonary endothelial cell but also smooth muscle cells that might exist at different locations in the pulmonary vasculature. Exploring endothelial cell populations and their functions in homeostasis and after injury will be an important research focus in coming years. It is not known whether the pulmonary vascular wall contains resident smooth muscle cell progenitors, nor is it known whether circulating bone marrow-derived progenitor cells have a role in mediating lung vascular repair or remodeling.

## REPROGRAMMING STEM CELLS (INDUCED PLURIPOTENT STEM AND EMBRYONIC)

The use of embryonic and induced pluripotent stem (iPS) cells to regenerate solid organs has great potential, but has not been well studied in lung. Further study using both pluripotent stem cell strategies is needed. A respiratory epithelium has been developed using mouse embryonic stem (ES) cells cultured as embryonic bodies with specific growth hormones in an air-liquid interface [33], and mouse ES cells in appropriate culture conditions can express cell markers of alveolar type II cell differentiation [34]; however, it is unclear whether these reprogrammed cells have functional potential. Application of murine ES models using human tissue is limited by ethical and technical challenges. Recent reports of the use of somatic cells dedifferentiated to embryonic-like cells raise the possibility of creating organ-specific autologous stem cells for therapy. Caution exists, however, because the dedifferentiation of these cells also makes them tumorigenic. Perhaps directing cell reprogramming without reversion to a pluripotent stem cell state of one lung cell type into another using defined factors to reconstitute the airway epithelium will be another strategy as has been accomplished for the pancreas [35]. Human amniotic fluid has recently been shown to contain a new source of stem cells that can be differentiated into lung epithelial cell lineages [36]. It is still not known whether “conversion” is restricted to gene and/or marker expression rather than functional potential. It should also be pointed out that differentiation of such cell populations into lung lineages in vivo (where measured) is generally minimal. Unfortunately, little is known about the appropriate signals to direct differentiation of multipotent stem/progenitor cells into respiratory epithelium and endothelium and to program cells to bioengineer an organized, complex, functional structure of the lung. Three-dimensional tissue engineering technologies may offer new strategies for investigating lung progenitor/stem cell interactions and function.

### Repair/Regenerative Capacity

Whereas turnover of the respiratory epithelium in the steady state is very slow, it has significant reparative capability after injury. The lung is exposed to many environmental irritants that can induce inflammation and injury. If the host response is appropriately controlled, local repair of the epithelium is possible. The lung filters 8,000-9,000 liters of air each day. When gas is exchanged, the respiratory epithelium must defend itself against irritants such as viral particles, tobacco smoke, pollutants, and aspirated food particles.

Endogenous stem cells probably play an important role in repair after injury, and depending on the compartment of the tracheobronchial tree that is injured, there will probably be different strategies for repair: basal cells in the trachea, variant Clara cells or BASCs in the bronchioles, and alveolar type II cells in the air sacs. One strategy for lung repair may include identifying molecules that can stimulate endogenous stem cell responses. Activation of canonical Wnt signaling in the niche containing BASCs can significantly increase the pool of stem cells [34]. Gata6 is required for these stem cells to differentiate and regenerate the respiratory epithelium. β-Catenin signaling in the developing lung attenuates stem cell differentiation, increasing self-renewal of the stem cell population [31]. An understanding of the controlled differentiation into functional respiratory epithelial cell populations to carry out gas exchange and airway defense will also need to be considered in strategies that attempt to harness the regenerative capacity of the endogenous stem cell. In addition, bone marrow-derived mesenchymal stem cells (MSCs) show promise and have been used to ameliorate lung injury in several models of lung disease. Although MSCs have stem cell properties and differentiation potential, the functional improvements seen in models of MSC therapy for lung injury, does not require engraftment or differentiation, suggesting that anti-immune, anti-inflammatory cytokine modulation, and/or stimulation of endogenous progenitor/stem cells are involved [37–39].

The report of a workshop on lung stem cell biology and disease, cosponsored by the NHLBI and the Cystic Fibrosis Foundation, is another excellent resource for information on current knowledge and controversies regarding lung stem/progenitor cells, including the potential therapeutic role of adult bone marrow-derived stromal cells in lung diseases [4]. Manipulating the apparent reparative functions of these cells is an area of keen research interest. Disorders such as cystic fibrosis involving defects in ion transport of airway epithelial cells, obstruction, and epithelial injury from recurrent infection, could be repaired with gene replacement using bone marrow stem cells. It is not clear whether the very low engraftment rates [40] will be sufficient to correct cystic fibrosis ion transport defects that contribute to impaired host defense and decrease the frequency of bronchopneumonias that ultimately lead to lung destruction and respiratory failure.

Injury to the alveolar compartment and aberrant repair contribute to the pathogenesis of idiopathic pulmonary fibrosis. Alveolar type I cells (AECI) cover 96% of the gas exchange surface area of the lung and are very fragile because of their very thin structure. Alveolar type II cells (AECII) that occupy only 4% of the gas exchange surface are thought to be responsible for alveolar type I cell regeneration after injury. It is not clear whether all alveolar type II cells can regenerate themselves and the AECI after injury or if only a subpopulation of AECII have this reparative capacity. In bone marrow transplant protocols when the alveolar compartment is severely damaged, donor hematopoietic stem cells (HSCs) have been found in the recipient lung, suggesting that the HSCs might contribute to proliferation of alveolar epithelial cells [41–43]. This finding has been refuted by Kotton et al. [44] as an artifact of autofluorescence. Idiopathic pulmonary fibrosis may be due to abnormal repair of the alveolar compartment and include the abnormal recruitment of bone marrow-derived fibroblasts after injury [45]. Clinical trials using stem cells for the treatment of lung diseases have not yet established safety and efficacy.

Osiris Therapeutics, Inc. currently has one product, Prochymal, an intravenously administered formulation of mesenchymal stem cells, in a phase II clinical trial for the repair of lung tissue in patients with moderate to severe chronic obstructive pulmonary disease (http://www.osiristx.com/clinical_trials_prochymal_copd.php). Results from this trial on safety and efficacy endpoints at 2 years in subjects with COPD have not yet been reported.

The complex structure and multiple functions of the lung compartments leave many unanswered questions in lung stem cell biology for the treatment of human diseases.

## GAPS

The molecular profile of the multipotent progenitor cells that reside at the various germ layers and regions of the lung as the respiratory tract forms is largely unknown [46]. Identification of stem cell compartments, responsible for repair of tracheal, bronchial, bronchiolar, and alveolar epithelium will be important. The endothelial progenitors and signals for their interaction with mesenchymal and epithelial tissue to permit reconstitution of an air exchange unit have not yet been determined. Although some specific molecular signals that are required to retain a stem cell niche have recently been identified, what signals exist in the steady state versus after injury need to be further elucidated. In addition, the molecular signals that permit repopulation of the epithelium and differentiation to specific lung cells with appropriate functional properties need to be further understood.

There are many questions that would be important to answer regarding lung stem/progenitor cell biology: What is the role of HSCs or MSCs in lung repair/regeneration and is engraftment necessary? Are there circulating factors and other mechanisms that induce endogenous progenitor cells to respond? Can the potential harmful effects of exogenous stem cell therapy be minimized to permit restoration of lung tissue? What are the signals released by the injured or remodeling lung that mobilize and attract bone marrow-derived stem cells to the site of injury? Can embryonic or induced pluripotent stem cells be programmed to reconstitute a functioning lung unit? How do cytokines and growth factors influence the function of stem cells and their progeny in acute and chronic lung disease? What are the biochemical and functional phenotypes of pulmonary endothelial and smooth muscle progenitor cells? What is the role of endothelial progenitor cells in vascular development, homeostasis, and repair after vascular injury? What role do these cells play in vascular disease?

Very limited research in tissue engineering of the lung is currently funded at NHLBI. In the short term tissue engineering may be used to enhance drug testing by developing three-dimensional in vitro models of disease that more closely reflect the in vivo state than current two-dimensional cell culture systems. Regenerative therapeutics to replace a large airway in a human has recently been demonstrated [47], but building a functional lung unit to replace defective lung tissue or lost lung tissue will require a longer time frame and multidisciplinary collaborations. Selection of the ideal cell source, strategies for optimal cell isolation, identification of a biocompatible and degradable scaffold, and choice of cell culture conditions and growth factors needed to direct differentiation of the diverse cell types are several aspects that remain to be determined.

## OPPORTUNITIES FOR RESEARCH SUPPORT

The NHLBI has a strong interest in supporting stem cell research and has encouraged growth in this area with several new Requests for Applications (RFAs) offered in 2007-2009 (Table 1). These RFAs support the goals of the NHLBI strategic plan, which highlights the goals of improving understanding of the molecular and physiologic basis of health and disease and developing personalized preventive and therapeutic regimens for cardiovascular, lung, and blood diseases [48]. Integrating advances in stem cell biology and regenerative medicine to develop clinically feasible applications is a long-term goal the NHLBI is working toward reaching in the future. Examples of past and current programs in heart, lung, and blood diseases are presented in Table 1 as well as resources for investigators.

**Table 1:** NHLBI/NIH supported stem cell initiatives

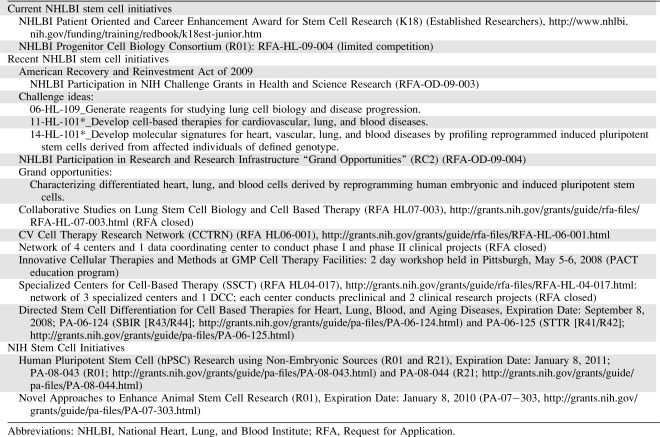

At present, there are a number of promising discoveries, technologies, and reagents that are poised to have a catalytic effect on the field of regenerative biology and medicine: the ability to constitute pluripotent stem cells from reprogramming with specific transcription factors using integrating viruses, nonintegrating viruses [49], or recombinant proteins [50]; and lineage tracing with Cre recombinase models [24,32], imaging of live cells [51], and markers of lung stem/progenitor cells [24,29,52]. In 2008-2009, NHLBI issued an RFA “NHLBI Progenitor Cell Biology Consortium.” The aim of this initiative is to markedly accelerate progress by forming a coordinated consortium that includes leading scientists in the field of cardiovascular, pulmonary, and hematopoietic cell biology, working closely with experts in the general field of progenitor cell biology. The goal of the consortium will be to harness advances in stem and progenitor cell biology to improve the understanding and treatment of cardiovascular, lung, and blood diseases. Areas of emphasis are expected to include lineages derived from murine and human embryonic stem cells, progenitor cells, somatic stem cells, and iPS cells. In addition, the derivation of iPS cells from patients with genetic diseases and differentiation of these cells to specific cell types are expected to give unique mechanistic insight into disease processes.

NHLBI recognizes that fundamental knowledge of pulmonary stem and progenitor cell biology, including progenitor cell lineages, lags behind the level of understanding in the cardiovascular and hematopoietic fields and has supported scientific meetings for lung stem cell biology and targeted funds through RFA mechanisms to support this new field (Table 1). An area of emphasis in recent initiatives is the generation of well-characterized lung progenitor cell fate maps, integrated with specific approaches to identify, purify, renew, and direct the differentiation of specific lineages of interest. Another important focus will be regional and/or developmental differences in cell origin and lineage, the identification of appropriate phenotypic markers to facilitate these studies, and programming and regeneration of three-dimensional structures for gas exchange and host defense. Research support of investigator-initiated and/or targeted RFAs is expected to foster innovative new technologies, reagents, strategies, and protocols that will have a major impact on scientific, translational, and clinical utility of lung progenitor cell lineages.

Investigators are encouraged to pursue careers in this exciting new area of lung biology that presents many opportunities for advancing the understanding of lung health and disease and developing new strategies for treatment (Table 2). We encourage investigators to contact staff, who can answer questions about NIH-supported research and review the NHLBI strategic plan [48]. Advancing lung stem cell biology will require multidisciplinary teams and a long-term effort to unravel the biologic processes of stem cells in the lung. Although no clinical research in lung stem cell therapies is currently funded by NHLBI, the knowledge gained by understanding the basic biology of the lung stem cell populations will be needed to translate to diagnostic and therapeutic strategies in the future. Several studies are being conducted outside the United States using cell therapy to treat lung disorders [5]. It will be important to be prudent, rigorous, and careful as researchers in the United States proceed in translational science in lung stem cell therapies. Early interactions with the Food and Drug Administration are encouraged to put in place the appropriate studies for investigational new drug and device applications.

## TRAINING

Preparing and training scientists in lung stem/progenitor cell biology will be important to accelerating the field. NHLBI supports multidisciplinary training of postdoctoral fellows through training grants and supports funding of educational symposia on fundamental stem cell biology at society meetings [5]. We encourage today's stem cell experts to identify promising Ph.D. and M.D. candidates and mentor their scientific careers. The T32 and F32 support mechanisms from NHLBI have supported many young investigators in pulmonary research, which often has led to the K series of awards for postdoctoral trainees. The Career Enhancement Award for Stem Cell Research enables investigators to acquire new research capabilities in the use of human or animal embryonic, adult, or cord blood stem cells (Table 1). This K18 award was specifically developed for candidates who have a clinical or research doctoral degree and are actively engaged in research of interest to the NHLBI. All candidates must have a sponsor, either within their own or at another institution, who is a well-qualified stem cell expert and will serve as a mentor.

There are >500 fellows in Pulmonary and Critical Care Medicine training programs [53]. A pipeline of pulmonary clinicians will be needed to prepare for the ultimate goal of regenerative medicine for a multitude of lung disorders of inadequate growth (bronchopulmonary dysplasia and congenital malformations) and injury (asthma, cystic fibrosis, acute lung injury, pulmonary fibrosis, and emphysema, and others).

## RESOURCES

The Production Assistance for Cellular Therapies (PACT) program supported by NHLBI was designed to develop novel somatic cellular therapies that will aid investigators by providing support in areas ranging from basic science through animal studies to proof-of-principle and eventually human clinical trials (Table 2). The cell-processing facilities provide the cellular product requested by an investigator along with the assurance that it is of clinical grade and is produced in a manner compliant with all regulatory requirements. In addition to product manufacture, PACT provides technical and regulatory expertise and educational programs. The Human Cell Therapy program provides Good Manufacturing Practice-grade cell resources to facilitate the development of new cell therapy. For additional information on NHLBI resources for cell-based therapy, see the editorial by John W. Thomas [54].

For those interested in reprogramming strategies for lung regeneration, human embryonic cells that are approved for NIH supported research are available through the National Embryonic Stem Cell Bank (Table 3). NIH supported investigators who propose to use human embryonic stem cells must comply with NIH policy, which is being revised and will be issued later this year.

**Table 2:** Research opportunities for lung stem/progenitor cell research

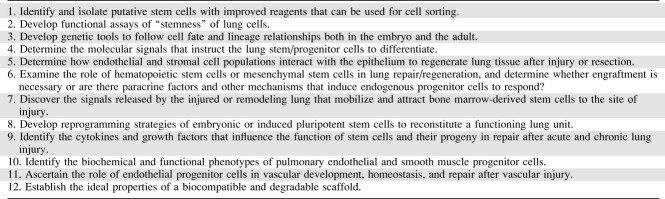

**Table 3:** Current NIH resources and policy for stem cells